# A facile method of treating spent catalysts via using solvent for recovering undamaged catalyst support

**DOI:** 10.1371/journal.pone.0296271

**Published:** 2024-01-02

**Authors:** Zaheer Abbas, Simon MoonGeun Jung

**Affiliations:** Green Carbon Research Center, Korea Research Institute of Chemical Technology, Daejeon, Korea; Saveetha Institute of Medical and Technical Sciences: Saveetha University, INDIA

## Abstract

The process of washing and removing crude oil from spent catalysts is a serious issue in both catalyst regeneration and precious metals recovery. In this work, five different solvents with various polar and aromatic properties were chosen to evaluate their impact on the catalyst support structure and crude oil recovery from oil-contaminated spent catalysts. After the deoiling process, the spent catalyst was analyzed by scanning electron microscopy, X-ray diffraction (XRD), Fourier transform-infrared spectroscopy, elemental analyzer, contact angle measurement, gas chromatography-mass spectrometry, inductively coupled plasma-atomic emission spectroscopy, and Brunauer Emmet Teller (BET) method. Our findings demonstrate that *p*-xylene and kerosene are more effective in removing oil than other solvents. This is due to crude oil’s similar polarity and molecular nature with kerosene and *p-*xylene. Considering the economical reason, kerosene is a better choice for deoiling spent catalyst compared to *p*-xylene as it is more affordable than *p*-xylene. XRD data show that the structure of the catalyst support was unaltered by the solvent treatment process, while BET data reveals that the surface area and pore volume are significantly enhanced after the deoiling process. These results imply that deoiling is a very crucial step for the recycling, regeneration, and reuse of spent catalysts. Our work is significant in developing sustainable approaches for managing spent catalysts, and minimizing waste and environmental pollution.

## Introduction

Catalysts have proven crucial to refine petroleum and produce fuel and other valuable products [1–4]. The main solid waste product of the refinery industry is the spent catalyst (SC), which contains a variety of hazardous metallic materials as well as potentially dangerous non-metallic constituents, for instance, carbon, elemental sulfur, and oils. Disposing or discharging the spent hydrodesulfurization (HDS) catalysts has been one of the reasons for causing industrial pollution in petroleum refineries. On the other side, these spent catalysts contain high-value metal components, which stem from crude oil or the catalyst itself. Therefore, the treatment of the spent desulfurization catalysts has attained importance because of not only the recovery of valuable resources but also waste management issues due to environmental regulations. Therefore, spent catalysts were highly regulated as hazardous waste materials in most countries, for example, the packaging, labeling, and shipping of SC. The disposal of spent catalysts is a significant environmental concern due to the presence of heavy metals, and other toxic organic and inorganic components. Spent catalysts (SCs) normally contain over 20% oil, and other metal contents, which can be recycled to reduce wasting resources and pollution [5–7]. The heavy oil hydrodesulfurization (HDS) process removes impurities like sulfur and nitrogen from crude oil through the HDS catalytic process [8–10]. After a pre-determined period of extensive use, these catalysts degrade to become spent catalysts as a result of metal toxicity, carbon deposition, and sintering of the active phase.

It is estimated that more than 10,000 tons of SCs have been generated globally every year [11–13]. As catalysts obtained many oil fractions and deposition of impurities during the refining process, SCs became hazardous solid waste and should be handled in a safe manner. A disposal method generally includes either landfilling them in a specific sealed container or transferring them to a recycling facility to obtain or regain precious resources [7]. Regardless of the method used for disposing of spent HDS catalyst, deoiling is important, because the residual oil in SCs contains hazardous components such as polychlorinated phenols (PCPs), polychlorinated biphenyls (PCBs), and polycyclic aromatic hydrocarbons (PAHs). When SCs were treated properly with various solvents, the pollution of the natural environment can be significantly reduced during the landfill process. During the metal recovery process, the presence of crude oil residue on the surface of SCs interferes with the contact between the catalyst surface and the solvent, which could influence the chemical leaching process for recovering valuable and important metals [14]. The activation and regeneration of catalysts are the first steps in the treatment of used spent catalysts. Those that cannot be activated or regenerated are often impregnated under different conditions to recover precious resources [15, 16]. Prior to the metal recovery process, the deoiling process is necessary because the surface of spent catalysts covered with oil will affect the leaching and extraction process during metal recovery. In the early days of petroleum refining, hot water was used to remove the oil-contaminated soil. Later, to remove oily soil, researchers employed a high-pressure water column with a powerful shear flow [17]. Despite the fact that the oil removal efficiency of used hydrotreating catalysts was close to 90%, a large quantity of oily wastewater was also generated [18]. To improve the pyrolysis efficiency of emitted oily sludge, various adsorption technologies have been applied [19]. However, these methods have encountered a number of issues including secondary pollution, insufficient oil removal, and high energy consumption [20, 21].

In recent years, more advanced technologies have been developed to remove hazardous organic chemicals and crude oil that are adhered to the surface of porous solid particles. For instance, the cost-effectiveness of the roasting method makes it widely used for the disposal of SCs. However, the major drawback of this process is the high possibility of flammability and an explosion unless the crude oil is completely removed. Additionally, unwanted impurities formed during the roasting process for example, NiMoO_4_ or CoMoO_4_ affect the post-treatment process [22–24]. Heat treatment, which is inexpensive and suitable for large-scale processing, is the most used process for oil removal from spent HDS catalysts [25]. However, a downside of combustible oil degradation by heat treatment is difficulty in temperature control. Additionally, incomplete burning of the oil residue in SC releases poisonous and harmful fumes to the environment. Mechanical methods are also utilized to effectively extract the oil residue from SCs. For example, a hydrocyclone is a type of cyclonic separator that separates the particles based on differences in gravity, which was used to remove contaminants from catalyst particles [26]. The use of sodium hydroxide and surfactant is currently employed as a quick and efficient way to remove oil from spent catalysts. The advantages of this approach include its low cost and ease of use. However, sodium hydroxide is extremely caustic and can harm equipment, while a higher temperature is also needed for the cleaning process. To overcome this issue and due to its environmental friendliness, the ultrasound-assisted hydrothermal method has been applied to improve the oil removal from the oil-contaminated spent catalysts [27–29]. Currently, new methodologies appearing on sustainable catalysts for the recovery of the catalyst. These methods encompass heterogeneous metal catalytic systems reactivity, selectivity, recyclability, and catalytic activity, as well as the mechanistic pathway of desired product formation, the removal of multiple organic dyes by using biomass-derived functionalized graphene aerogels [30–33].

Solvent extraction has received much attention recently for removing oil from spent HDS catalysts. Organic solvents such as naphtha [34], acetone [35–37], n-heptane [38], and toluene [39, 40] have been studied. The most notable benefit of the solvent extraction process is high oil recovery efficiency when performing catalyst remediation. Despite the fact that the solvent extraction method can effectively remove oil, it is unsuitable for safe industrial-scale applications since the solvents are very volatile and have a low flash point [12]. To overcome this issue, solvents with high boiling points and high flash points can be used to deoil spent catalysts. Recently, a detailed investigation on the removal of crude oil from SCs using several organic solvents was conducted [41]. Although solvent treatment of SCs has been widely explored, the effect of solvent extraction on catalyst structure and support has rarely been studied. Therefore, a quick, effective, and mild technique is still required to extract the oil residue from SCs.

In this work, to establish optimal solvent properties, five solvents such as n-hexane, kerosene, *p*-xylene, acetone, and dichloromethane, were adopted to compare polar and aromatic properties to remove oil residue, and also to investigate their impact on the catalyst support structure of the spent hydrodesulfurization (HDS) catalyst. The crude oil residue on spent catalysts and the applied solvents were successfully recovered. Both spent and the deoiled spent catalysts were characterized comparatively, which indicated that a considerable amount of metals and oil contents had been deposited on the surface. The spent catalysts (SCs) and the deoiled SCs were characterized by using a scanning electron microscope (SEM), energy-dispersive X-ray spectroscopy (EDXS), Fourier transform-infrared spectroscopy (FT-IR), X-ray diffraction (XRD), contact angle (CA) measurement, Brunauer-Emmet-Teller (BET), inductively coupled plasma-atomic emission spectroscopy (ICP-AES), gas chromatography-mass spectrometry (GC-MS), and elemental analysis (EA).

## Experimental

### Materials

The spent hydrodesulfurization (HDS) catalyst was a rod-shaped structure of 1 mm diameter and 4 mm length, which was provided by the Korean local petroleum corporation. Dichloromethane (~99.9%) and acetone (≥99.7%) were purchased from Duksan Chemical Co. (Korea). *P*-xylene (≥99%), n-hexane (≥99%), and kerosene (≥95%) were from Sigma-Aldrich.

### Experimental procedure

5 g of spent HDS catalyst and 30 mL of solvents were added to 100mL flasks. During the oil removal process, the flasks were capped to avoid loss of solvents and placed in an oil bath with continuous magnetic stirring. The mixture was heated to 50°C at the stirring speed of 500 rpm for 30 minutes. Then, the mixture of SCs and solvent was separated by filtration. The solid obtained by filtration was dried in an oven at 80°C for 24h. Solvents were removed by rotary evaporator and the recovered oil was obtained under vacuum, except for kerosene owing to the high boiling point range of 150–300°C. Kerosene could not be completely removed by a rotary evaporator. After drying, the crude oil was analyzed by gas chromatography-mass spectrometry (GC-MS).

Boiling points and their relative polarities of solvents are given in Table 1 [42], five grams of SC with 30ml of solvent were mixed and stirred at 500 rpm for 30 minutes, at 50°C respectively.

**Table 1:** Boiling point and relative polarity of solvents.

Solvent	Boiling point (°C)	Relative polarity
n-hexane	69	0.009
kerosene	150–300	N/A*
*p*-xylene	138.4	0.074
dichloromethane	39.6	0.309
acetone	56	0.355

*kerosene is a non-polar organic solvent.

The weight loss ratio was applied to evaluate the oil removal rate of the raw SCs and the deoiled SCs.

$$Weight\;loss\;ratio=\frac{Wo-W}{Wo}\times 100$$

where W_o_(g) and W(g) represent the mass of the raw SC and the deoiled SC respectively.

The oil removal efficiency of different solvents was also determined by liquid following the previous procedure and calculated [43].

$$Oil\;removal\;efficiency=\frac{Co-C}{Co}\times 100$$

where C_o_(g) and C(g) represent the mass of oil content in the raw SC and the deoiled SC respectively.

### Solvent selection and optimization

**Fig 1:**
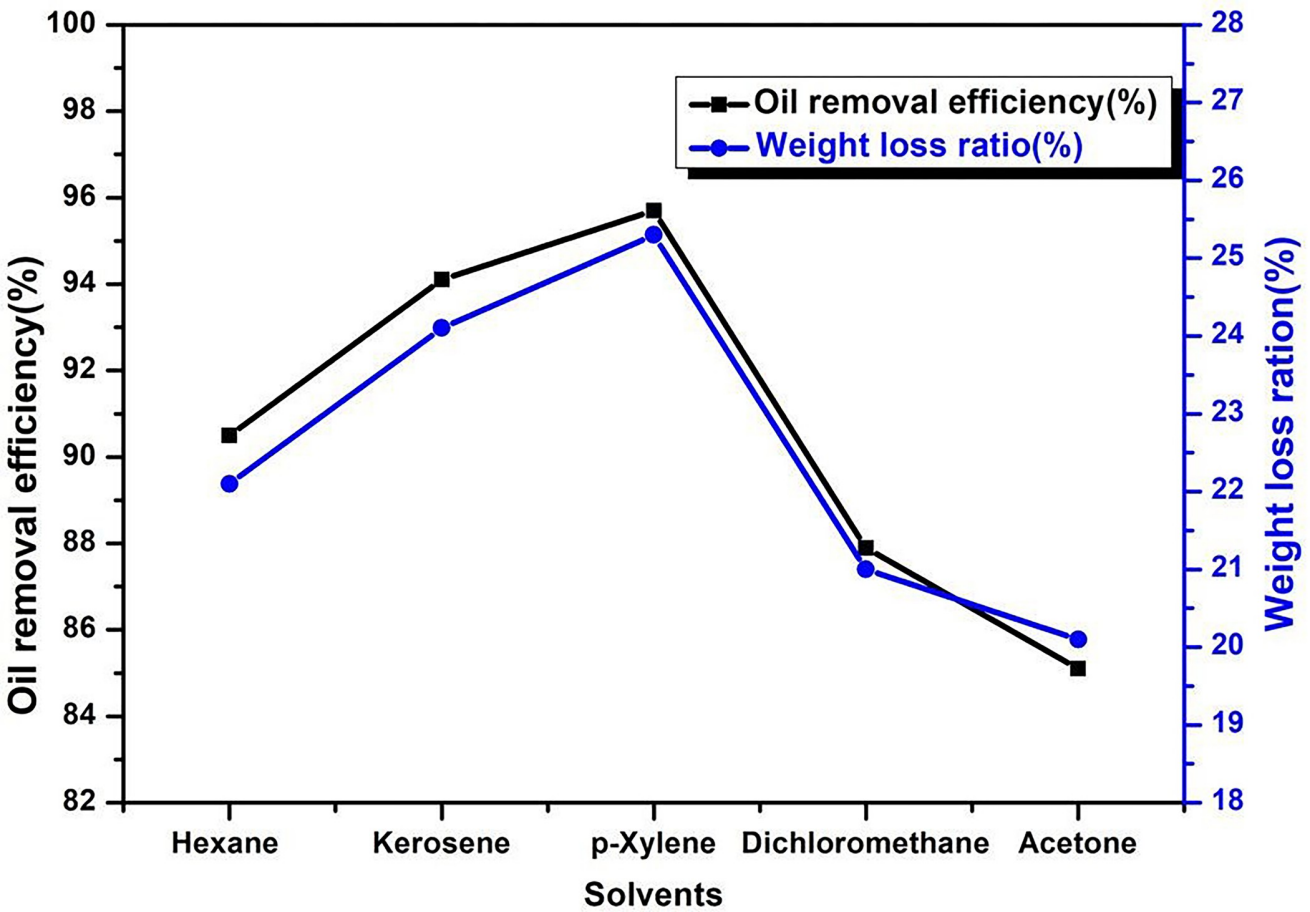
Oil removal efficiency of crude oil residue vs. weight loss ratio of SC.

The affinity between solvent molecules and the oil components is influenced by the polarity and aromaticity of solvents, which have an impact on the solvent’s ability to effectively remove the oil residue on SCs. Therefore, the selection process of the best affinity solvent to remove and extract the oil residue from SCs is important. In this work, five solvents with varying degrees of polar and aromatic properties were selected to extract the oil contents from SCs. Fig 1 shows the oil removal efficiency and weight loss ratio of HDS catalysts.

According to the solvent similarity and compatibility, as the majority of the oil residue in the spent HDS catalyst is non-polar, the less polar solvent has higher washing efficiency [44, 45]. Therefore, as solvent polarity increases, solvent washing efficiency gets lower. The SC washing efficiency in descending order was *p*-xylene > kerosene > n-hexane > dichloromethane > acetone. In the case of *p*-xylene, owing to aromaticity, although *p*-xylene is more polar, its oil-removing efficiency is higher than hexane. In terms of aromaticity, *p*-xylene is an aromatic solvent and it has similar molecular structures to crude oil components.

Furthermore, in regards to Fig 1, *p*-xylene and kerosene are more effective in removing oil from surfaces than other solvents. In terms of aromaticity, crude oil, kerosene, and *p*-xylene have similar polarities as well as similar chemical structures. The gas chromatography-mass spectrometry analysis (GC-MS) also confirmed that the aromatic fraction of the recovered oil contained chemical compounds possessing a similar and comparable structure to that of *p*-xylene, resulting in enhanced extraction efficiency of the oil residue in SCs.

However, from an economic standpoint for large-scale treatment, kerosene is preferred rather than *p*-xylene for deoiling SC, because it is less expensive and readily available. From Fig 1 and Table 2, it was found that the trends in weight loss of SC before and after solvent treatment, are the same as the washing efficiency. For instance, the weight loss of SCs is higher after *p*-xylene and kerosene treatment compared to what other solvents did. It was also noted the weight loss higher than the amount of oil residue in SCs is due to the removal of other deposited resources on the surface of SCs, other than oil residue.

**Table 2:** Oil contents recovery vs. weight loss ratio.

SC treated with solvent	Oil contents recovered (g)	Weight loss ratio (%)
SC treated with hexane	1.01	22.1
SC treated with kerosene	N/A*	24.1
SC treated with *p*-xylene	1.2g	25.3
SC treated with dichloromethane	0.97	21.0
SC treated with acetone	0.90g	20.1

* Due to the high boiling point of kerosene, recovery of oil contents was incomplete.

### Characterization

An inductively coupled plasma-atomic emission spectrometer (ICP-AES, Thermo Scientific iCAP 6500 duo) analysis was conducted to identify the main components of SC. The morphologies of the raw SC and the deoiled SC were characterized by scanning electron microscopy (SEM, XL30S FEG, Philips) with Schottky field emission as an electron gun at an acceleration voltage of 10 kV after platinum coatings. SEM is also equipped with an energy-dispersive X-ray spectroscopy system (EDXS, Tescan MAGNA FEG SEM) accelerating voltage of 5 kV with a platinum coating of Quorum Q150T ES / 10 mA 120s.

Fourier transform-infrared spectrometer (FT-IR) was used to characterize the functional groups of samples. X-ray diffraction (XRD) analysis was operated via XRD (Ultima IV, Rigaku) with Cu Kα radiation (λ = 1.5 Å), using a generator voltage of 40 kV and a current of 40 mA, and a step of 0.04° at a rate of 2° per minute in the range from 5 to 80°.

We examined the pore volume and surface area of catalyst samples through the nitrogen adsorption Brunauer-Emmett-Teller (BET, Nova 2000, quanta chrome) method. The contact angles of SC were measured by the contact angle analyzer (Phoenix—300 Touch, SEO, Korea) [46]. A gas chromatograph-mass spectrometer (GC-MS, Agilent 6890N gas chromatograph– 5973N mass selective detector) was employed to analyze the recovered crude oil from the deoiled catalyst. A flow rate of 1 mL/min of high-purity helium was used as the carrier gas. Based on an automatic library search of the NIST library version 2.0, chromatographic peaks were identified. (National Institute of Standards and Technology, U.S. Department of Commerce).

## Results and discussion

An inductively coupled plasma-atomic emission spectroscopy (ICP-AES) was used to identify the main components of the spent HDS catalyst. The chemical composition of SC by ICP-AES was listed in Table 3.

**Table 3:** Chemical composition of spent HDS catalyst.

Elements	S	Al	Ni	Co	V	Mo
**Mass fraction (%)**	10.4	18.2	2.85	0.007	8.16	3.28

**Fig 2:**
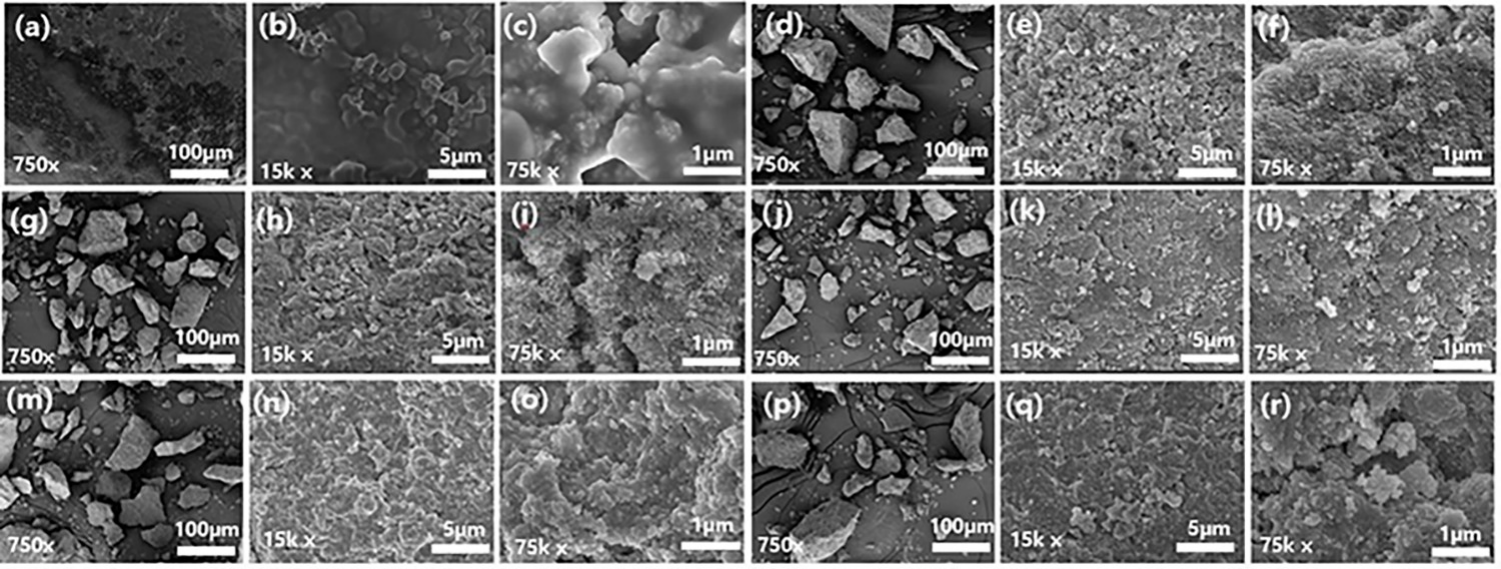
SEM images at different magnifications: (a)-(c) raw SC; (d)-(f) SC by hexane treatment; (g)-(i) SC by kerosene treatment; (j)-(l) SC by *p*-xylene treatment; (m)-(o) SC by dichloromethane treatment; (p)-(r) SC by acetone treatment.

SEM was used to determine how SCs differed in their morphology and structural characteristics before and after being treated with solvents (Fig 2). SEM images of the raw SC with various magnifications are shown in Fig 2(A)–2(C). It was observed that the oil residue on the raw SC is poorly dispersed in Fig 2(A). The catalyst surface is completely covered with oil in Fig 2(B) and 2(C). Fig 2(D)–2(R) display the SEM images of SC after solvent extraction at various magnifications.

As a large number of pore structures were exposed on the surface of the deoiled SC, the fresh HDS catalyst was able to adsorb a significant amount of crude oil throughout the desulfurization and hydrogenation process. As Fig 2(F), 2(I), 2(L), 2(O) and 2(R) show, the structures of alumina support loaded with metal were also exposed after being treated with solvents.

**Fig 3:**
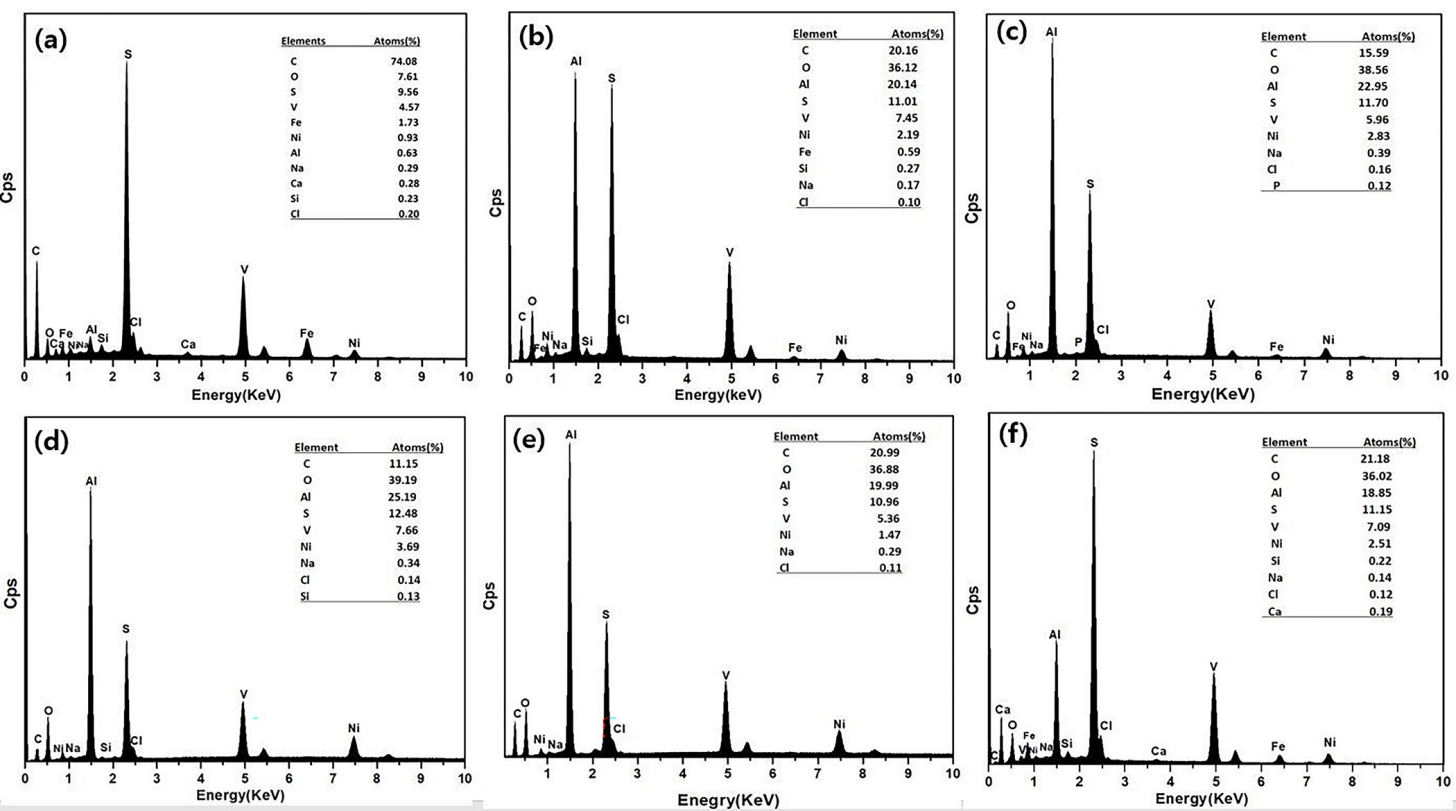
EDX analysis: (a) raw SC, (b) SC treated by hexane, (c) SC treated by kerosene, (d) SC treated by *p*-xylene, (e) SC treated by dichloromethane, (f) SC treated by acetone.

The elemental compositions of the spent HDS catalyst, prior to and following solvent treatment were characterized by using energy dispersive X-ray spectroscopy (EDX) in Fig 3(A)-3(F). Typically, HDS SCs are loaded with active metals like Ni, Co, and Mo on alumina support [5]. The deoiled SC should therefore have components such as Al, O, Ni, and V, while V was deposited from crude oil in Fig 2(A). Owing to the presence of crude oil in the HDS SC, the surface of SC is entirely covered by crude oil, which mainly comprises C, O, Ca, S, and Fe.

After solvent treatment, C, O, Ca, and Fe were greatly decreased, demonstrating that the adsorbed oil contents on the SC were successfully extracted from SC. On the other side, Al, Ni, O, and other metal content increased considerably, as shown in Fig 3(B)-3(F). This is a result of the active metal being completely exposed, following the removal of oil residue on SCs.

Elemental composition by elemental analysis (EA) was conducted as listed in Table 4, which indicates the elemental composition of C, N, H, and S of the raw SC and the deoiled SC. It is confirmed that crude oil residue on SC was effectively removed by solvent extraction as the carbon contents are significantly reduced in the deoiled SC. It was noted that SC treated by *p*-xylene and kerosene contains the lowest percentage of carbon, which results in comparably the most effective solvents. The results of EA agree well with those of EDX analysis, which proposes that kerosene and *p*-xylene effectively remove oil from SC compared to hexane, acetone, or dichloromethane. Furthermore, similar to EDX, Elemental analysis (EA) also revealed that sulfur content increased after the removal of oil from the spent catalyst. The significant increase in sulfur content observed after solvent treatment can be attributed to the catalyst surface being exposed during the oil removal process. Sulfur compounds during the oil refining process on the surface of the spent HDS catalyst were removed by solvent treatment. However, the HDS catalyst intrinsically contains sulfided Mo such as CoMoS and MoS_2_. During the deoiling process, as the solvent effectively removes the oil and impurities, it also has the capacity to strip away sulfur compounds that have adhered to the catalyst’s surface. This leads to the release of sulfur species, contributing to the observed increase in sulfur content in the analyzed samples.

**Table 4:** Elemental analysis of raw SC and deoiled SC.

Catalyst	Carbon (%)	Nitrogen (%)	Hydrogen (%)	Sulfur (%)
The raw SC	26.17	0.19	3.99	11.06
SC treated with hexane	10.36	0.24	1.19	11.36
SC treated with kerosene	7.52	0.20	1.50	12.04
SC treated with *p*-xylene	6.47	0.19	1.04	12.82
SC treated with dichloromethane	10.98	0.17	0.98	11.59
SC treated with acetone	11.05	0.18	1.58	11.52

**Fig 4:**
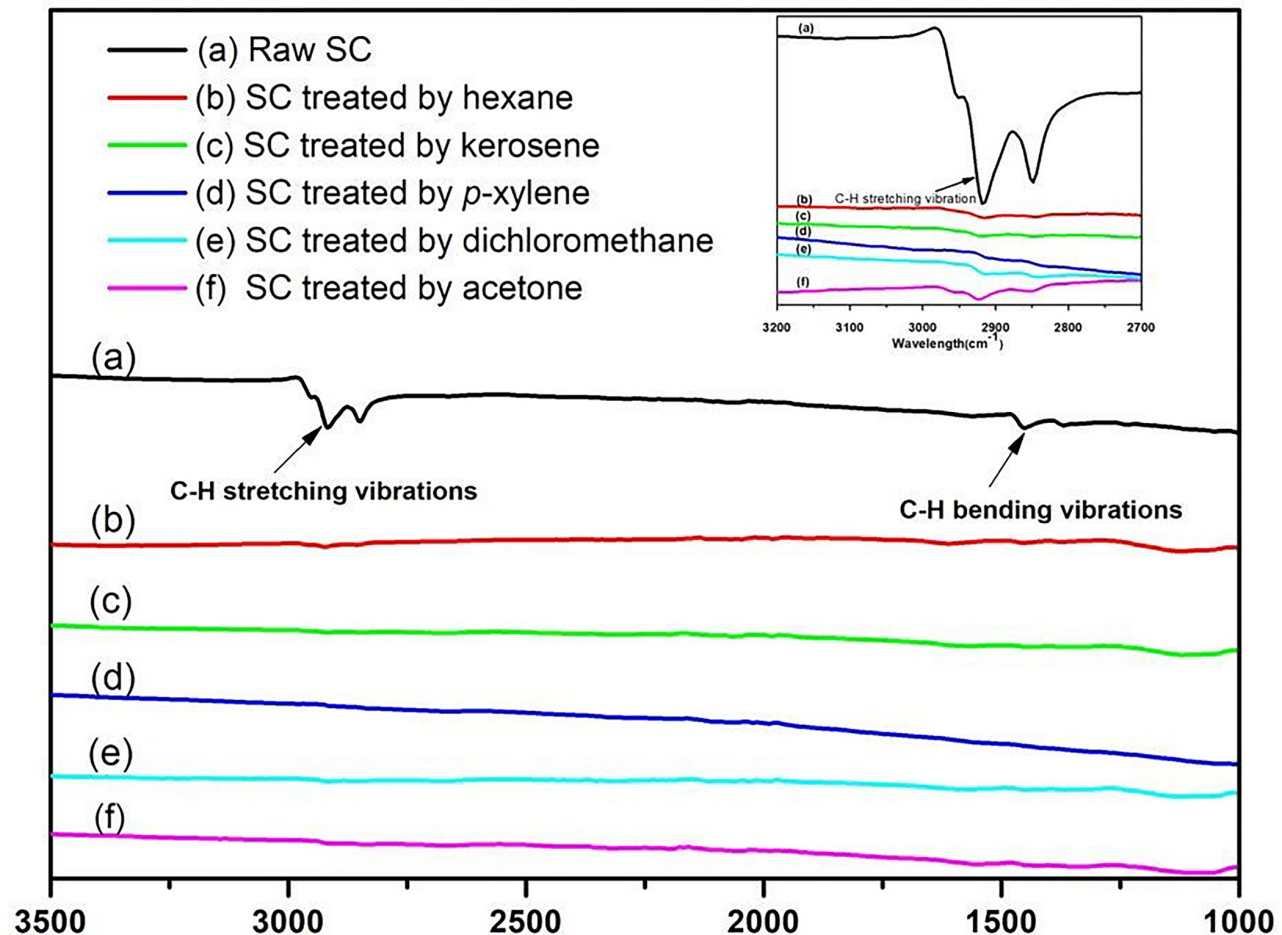
FT-IR spectra of the spent catalysts before and after solvent treatment: The inset figure of the enlarged part between 3200cm^-1^ and 2700cm^-1^.

FT-IR spectra before and after solvent extraction are shown in Fig 4. As SC contains hydrocarbons, FT-IR spectra have C-H stretching and bending peaks. The asymmetric and symmetric stretching vibrations of the carbon-hydrogen (C-H) bonds caused the peaks at 2851 cm^-1^ and 2954 cm^-1^ respectively. Another absorption band was observed at 1372 cm^-1^ and 1455 cm^-1^. These peaks are the result of asymmetric and symmetric bending vibrations of carbon-hydrogen bonds [47–49]. However, after being treated with five solvents, C-H symmetric and asymmetric stretching at 2851 cm^-1^ and 2954 cm^-1^, and bending vibration peaks at 1372 cm^-1^ and 1455 cm^-1^ of SC all disappear. It indicates that the contaminated oil from SC was rapidly and thoroughly removed during solvent treatment. As no additional peaks or bands appear after the treatment of SC with solvents, the structure of SC remains unchanged after solvent treatment.

**Fig 5:**
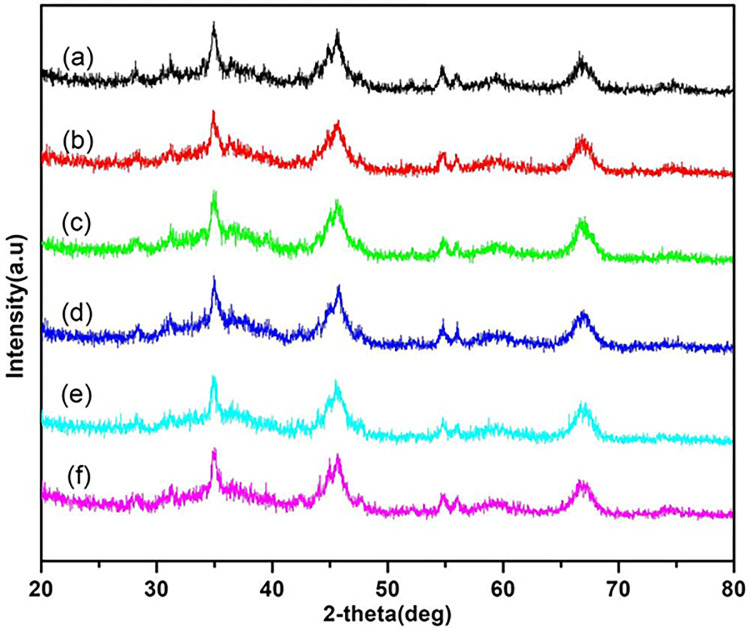
X-ray diffraction patterns of the spent catalyst: (a) raw SC, (b) SC treated by hexane, (c) SC treated by kerosene, (d) SC treated by *p*-xylene, (e) SC treated by dichloromethane, (f) SC treated by acetone.

The resource recovery process by solvent extraction process often causes detrimental effects on the structure of SC, which is not beneficial in the case of reusing, regeneration, and recycling of SC. Six XRD diffraction patterns in Fig 5 indicate that Al_2_O_3_ support peaks are located at diffraction angles 2θ = 46.9° and 15.7° [50–52]. The characteristic peaks at 35°, 54.5°, 56°, and 68.5° are completely intact after solvent extraction with five solvents. Hence, the XRD patterns demonstrated that the solvent treatment method did not modify or alter the lattice structure and geometry of SC and maintained the undamaged catalyst support.

**Fig 6:**
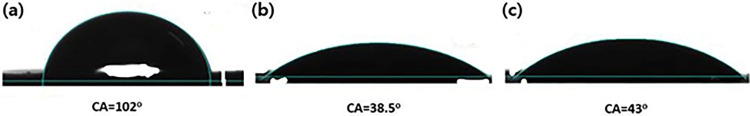
Contact angles of spent catalyst: (a) raw SC, (b) SC treated by *p*-xylene, (c) SC treated by acetone.

The contact angle (CA) between the probing liquid and the solid surfaces is the most frequently utilized factor to define the wettability of solids [46]. The desorption of oil residue on the surface of SCs will be considerably influenced by the wettability of the solid. The CAs were determined to study the surface properties and oil removal efficiency before and after solvent treatment of SC. Due to the presence of several hydrophilic elements on the catalyst surface, such as MoS and Al_2_O_3_, the deoiled SCs are expected to be hydrophilic [27]. Prior to solvent treatment, the CA of the used HDS catalyst was 102°, which implies that the catalyst surface had hydrophobic properties and had absorbed a significant amount of crude oil. However, the contact angle of the deoiled SC was 43° with acetone, and 38.5° with *p*-xylene in Fig 6. It shows that the oil residue was removed more effectively from SC by *p*-xylene, in comparison with SC treated with acetone.

The deposition of crude oil residue affects the pore structure to deactivate the process catalyst. These textural properties of SC before and after solvent treatment were analyzed by BET in Table 5. The surface area and pore volume of the raw SC before solvent treatment are very low at 2.44m^2^/g and 0.01 cm^3^/g. The primary reason for the loss of surface area and pore volume is the deposition of oil and organics on the surface and the pore mouth blocking of the catalyst during the petroleum refining process [53–55]. As Table 5 listed, when SCs were treated with solvents, the surface area and pore volume were noticeably enhanced, whereas the pore sizes were reduced. It is attributed to the removal of solvable compounds or unstable substances, which are present in the pores of SCs. In our work, *p*-xylene and kerosene treatment indicated enhanced surface area and pore volume, compared to other solvent treatments. The results from the BET analysis demonstrate that the spent catalysts, especially those treated with *p*-xylene and kerosene, exhibit comparable pore sizes and volumes to the pristine HDS catalysts. Therefore, BET data, substantiate our work on regenerating the spent catalysts and confirm that the treatment method has preserved the catalyst’s microstructure and active sites.

**Table 5:** Surface areas and porosities of SC.

	BET surface area (m^2^/g)	pore volume (cm^3^/g)	pore size (nm)
The raw SC	2.44	0.01	18.62
Fresh pristine catalyst	99.34	0.22	9.11
SC treated with hexane	80.43	0.20	11.20
SC treated with kerosene	90.25	0.25	12.09
SC treated with *p*-xylene	92.76	0.28	10.95
SC treated with dichloromethane	75.08	0.22	11.09
SC treated with acetone	70.14	0.21	11.37

**Fig 7:**
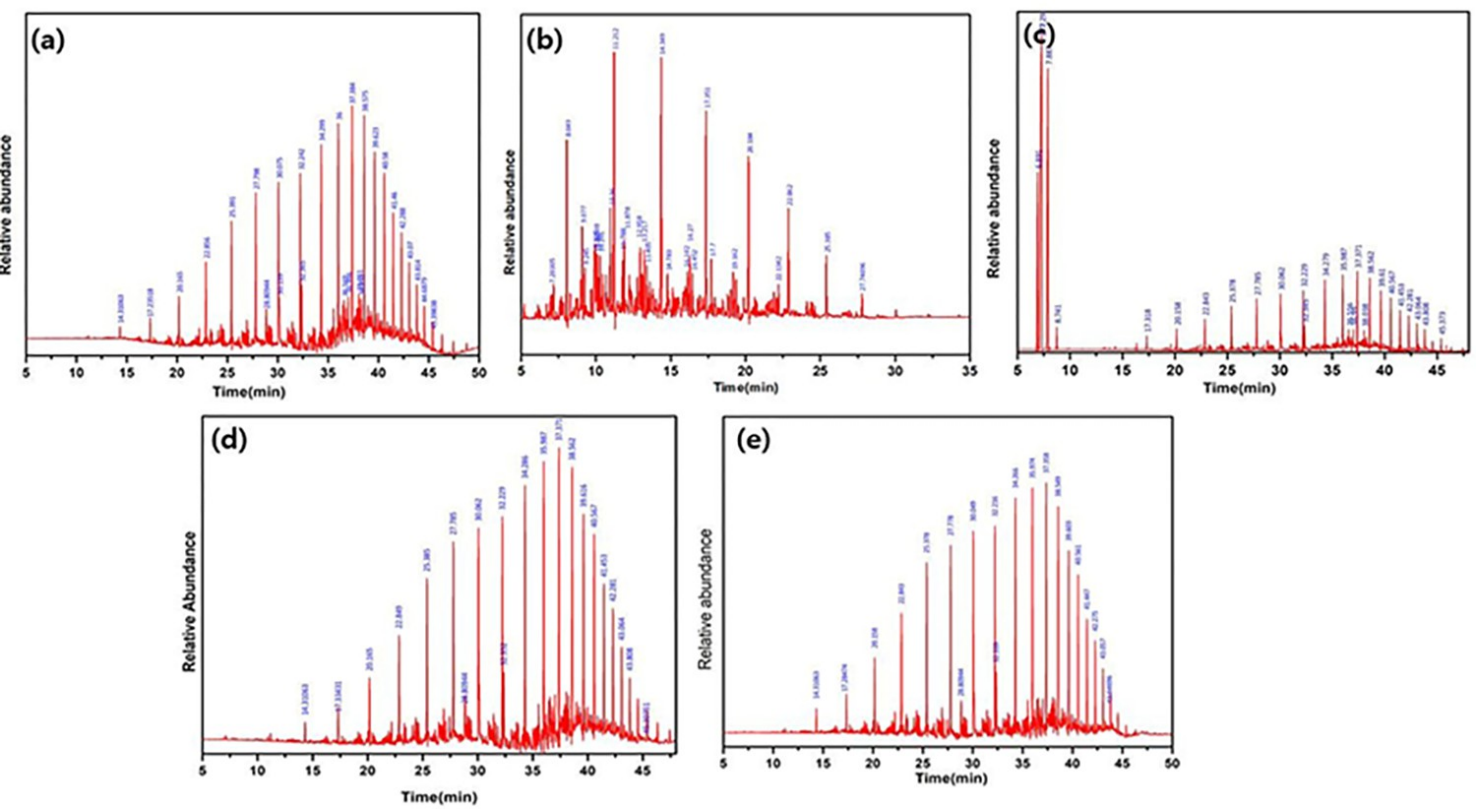
GC-MS spectra of recovered oil: (a) treated by hexane, (b) treated by kerosene, (c) treated by *p*-xylene, (d) treated by dichloromethane, (e) treated by acetone.

In the past, a few chromatographic methods were used to determine various components in petroleum and biofuel mixtures [56]. Thin layer chromatography with flame ionization detection (TLC/FID) was the initial technique used to track the transesterification process in oils [57]. This approach was found to be sensitive to humidity, displaying material inconsistencies despite its short analytical duration. To overcome such issues, high-performance liquid chromatography (HPLC) and gas chromatography (GC) techniques were developed subsequently [58]. In this work, GC-MS was employed to probe the constituents in recovered oil from SC. GC-MS chromatograms of the organics in the recovered oil are shown in Fig 7(A)-7(E). Table 6 presents the corresponding organic composition of the recovered crude oil from SCs. Based on the chemical composition of organic matter, a quantitative study of the organic content of various carbon atoms (Cn, = 1, 2, 3… 30) was carried out. *P*-xylene and kerosene were found to recover the majority of the challenging-to-recover heavy fractions of crude oil, such as C9 to C22 in a relatively short period of time, which is between 10 to 25 minutes. As Fig 7(B) and 7(C) indicate, oil fractions are removed quickly in the beginning, with kerosene and *p*-xylene, compared to hexane, acetone, and dichloromethane. It proposes that kerosene and *p-*xylene are well-suited for treating SC.

**Table 6:** Organic composition of recovered oil by *p*-xylene extraction.

RT	Possible Compound Name	Formula
**6.891**	Ethyl benzene	C_8_H_10_
**7.272**	*m*-Xylene, *p*-xylene	C_8_H_10_
**7.887**	*o*-Xylene	C_8_H_10_
**8.741**	Benzene, (1-methylethyl)-	C_9_H_12_
**20.158**	Tridecane	C_13_H_28_
**22.843**	Tetradecane	C_14_H_30_
**25.378**	Pentadecane	C_15_H_32_
**27.785**	Hexadecane	C_16_H_34_
**30.062**	Heptadecane	C_17_H_36_
**32.229**	Octadecane	C_18_H_38_
**32.345**	Hexadecane, 2,6,10,14-tetramethyl	C_20_H_42_
**34.279**	Nonadecane	C_19_H_40_
**35.987**	Eicosane	C_20_H_42_
**37.371**	Heneicosane	C_21_H_44_
**38.562**	Dococosane	C_22_H_26_
**39.610**	Tricosane	C_23_H_48_
**40.567**	Tetracosane	C_24_H_50_
**41.828**	Pentacosane	C_25_H_52_
**42.281**	Hexacosane	C_26_H_54_
**43.064**	Heptacosane	C_27_H_56_
**43.808**	Octacosane	C_28_H_58_
**44.552**	Nanocosane	C_29_H_60_
**45.373**	Triacontane	C_30_H_62_

A comparative analysis of the oil removal efficiency using the proposed method in this work, in comparison to previously reported methods, is shown in Table 7. It is worth noting that, while some reports in the literature have shown high oil removal efficiency, these cases frequently involved the use of ultrasonic assistance and treatments with sodium hydroxide, sometimes under harsh acidic conditions and elevated temperatures. However, it is crucial to emphasize that these alkaline and acidic conditions can be corrosive to the spent catalyst, potentially leaching valuable metals from its structure. In contrast, our method employs less corrosive organic solvents. Importantly, we have observed that our method’s oil removal efficiency is comparable to that of the previously reported methods, but with the added benefit of employing organic solvents that can be recycled. This not only underscores the economic viability of our approach but also highlights its capacity to maintain a less harsh and more environmentally friendly operational environment for spent catalyst regeneration.

**Table 7:** Oil removal efficiency comparison with reported methods.

	Method	Solvents	Temperature (°C)	Stirring time (h)	Oil removal efficiency (%)	Reference
1	Conventional heating and stirring	Alkaline SDS solution: (NaOH(aq)+ SDS)	90°C	4h	98%	[27]
2	Ultrasonication	Alkaline solvent: NaOH(aq)	75°C	3.5h	96%	[22]
3	Ultrasonication	Organic solvent: Ethanol(aq)	55°C	2h	98%	[5]
4	Conventional heating and stirring	Acidic co-solvent: Brij-58/1,2-dimethylbenzene	70°C	4h	93.5%	[41]
5	Conventional heating and stirring	Organic surfactant: Brij-58	40°C	4h	67.7%	[41]
6	Conventional heating and stirring	Organic surfactant: BS-12	40°C	4h	56.1%	[41]
7	Conventional heating and stirring	Organic solvent: Benzene	40°C	4h	89.8%	[41]
8	Conventional heating and stirring	Organic solvent: 1,2 dimethyl benzene	40°C	4h	91%	[41]
9	Conventional heating and stirring	Organic solvent: Cyclohexane	40°C	4h	87.3%	[41]
10	Conventional heating and stirring	Organic solvent: Cyclohexene	40°C	4h	86.9%	[41]
11	Conventional heating and stirring	Organic solvent: *p*-xylene	65°C	0.2h	93.5%	[14]
12	Conventional heating and stirring	Organic solvent: Kerosene	50°C	0.5h	94.5%	This work
13	Conventional heating and stirring	Organic solvent: *p*-xylene	50°C	0.5h	96%	This work

## Conclusion

The solvent extraction and treatment method have been widely used in many industrial processes. In particular, *p*-xylene and kerosene are highly effective for recovering and removing oil residue from spent catalysts, followed by hexane, dichloromethane, and acetone in order. The polarity and aromaticity of *p*-xylene and kerosene influence the solvent treatment efficiency as kerosene and *p*-xylene have comparable polarities and molecular structures, compared with crude oil. BET data shows that the surface area and pore volume of SC are significantly increased and possess similar pore size and volume with fresh catalysts after solvent extraction. The analysis of XRD patterns suggests that the solvent treatment processes did not alter the structure of SC and maintained the catalyst support undamaged. This reveals that these organic solvents can be used in large quantities without risk, compared to harsh acidic treatment. It is advantageous for the recycling or regeneration of used catalysts. The findings in this work are significant in terms of developing sustainable mild approaches for the management of spent catalysts and for minimizing waste and environmental pollution.

## Supporting information

S1 Graphical abstract (TIF)

S1 File (ZIP)
