# Supplementary figures and images for: A facile method of treating spent catalysts via using solvent for recovering undamaged catalyst support
Source: PLoS One. 2024 Jan 2;19(1):e0296271. doi: 10.1371/journal.pone.0296271 (PMC10760920; doi:10.1371/journal.pone.0296271)

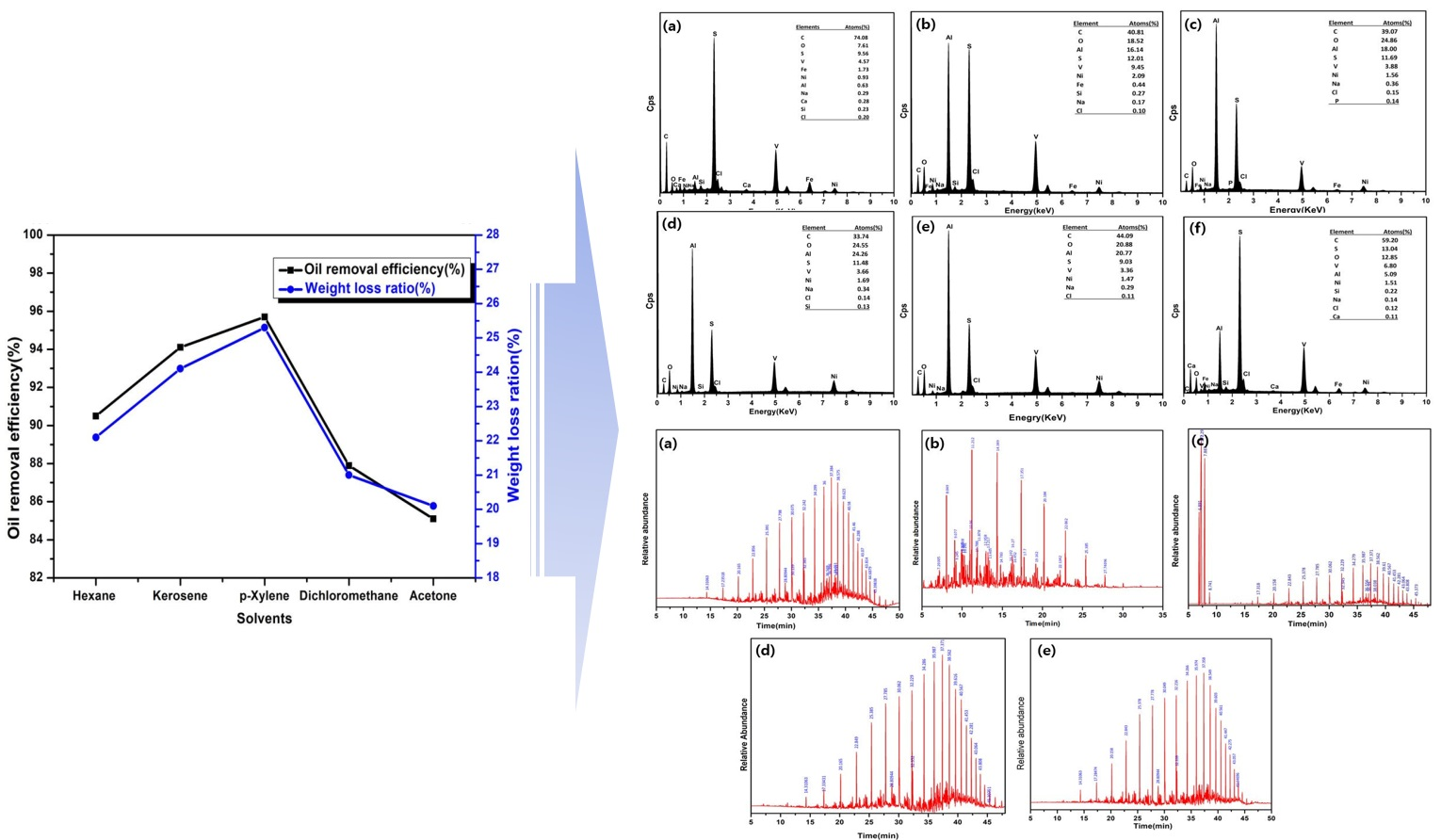

Supplement: S1 Graphical abstract — (TIF) [file pone.0296271.s001.tif]
